# Comparative treatment with hyperbaric oxygen therapy in a model of systemic loxoscelism in rats

**DOI:** 10.22038/IJBMS.2022.65357.14385

**Published:** 2022-12

**Authors:** Mireille Toledo-Blas, Antonio Franco-Vadillo, Selma A Somilleda-Ventura, Brenda Dominguez-Ruiz, Gustavo Guevara-Balcazar, Alexandre Kormanovski-Kovzova, Pedro Lopez-Sanchez, Rosa A Jarillo-Luna, Eleazar Lara-Padilla, Maria Carmen Castillo-Hernandez

**Affiliations:** 1 Sección de Estudios de Posgrado e Investigación, Escuela Superior de Medicina, Instituto Politecnico Nacional, México City, México

**Keywords:** Anti-oxidant therapy Cyclooxygenases Hyperbaric oxygen therapy Spider venoms, Systemic inflammatory-response

## Abstract

**Objective(s):**

Spiders of the *Loxosceles* genus, known as violin spiders, produce venom with dermonecrotic and systemic effects, as it is a species widely distributed in the world, its study represents a high medical relevance. Systemic loxoscelism, which occurs in 1 in 5 cases and is the most frequent in children, can be fatal, so the study of effective therapy is of great relevance. In the present study, we compared different therapeutic options to mitigate the systemic effects of *Loxosceles boneti* venom in a model in which prepubertal rats were used.

**Materials and Methods:**

A model of systemic intoxication by *L. boneti* venom was provoked in male Wistar rats. Study groups were formed: healthy control, with venom and untreated control, treatment with N-acetylcysteine, and/or hyperbaric oxygenation therapy. Subsequently, pathological analysis of the kidney and lung was performed. The oxidant-antioxidant response was evaluated, and molecular analysis of the COX-1 and COX-2 enzymes was performed.

**Results:**

Regenerative changes were observed at the cellular level in both treatments, being more noticeable in the hyperbaric oxygen therapy (HBO) group. The anti-oxidant response was outstanding in the same group.

**Conclusion:**

Both treatments offer considerable benefits, however; further studies are needed to provide adequate therapeutics.

## Introduction

The spider of the genus *Loxosceles* belonging to the family *Sicariidae* is also known as the brown recluse spider or violin spider, due to its characteristic violin-shaped marking on the cephalothorax (1). It is a widely distributed spider throughout the world (2). Mexico and USA are the countries with the highest number of species in the world (3); especially Mexico, which has the greatest diversity of *Loxosceles* species (4). The increase in exponential urbanization in much of the world has provoked in turn a relationship between native species and humans which leads to an increase in accidental attacks caused by this arachnid, which is considered of medical importance since in its bite are found enzymes such as sphingomyelinase D responsible for the dermonecrotic effects characteristic of this bite (5). Venom causes loxoscelism, a disease that can cause edema, vasodilatation, leukocyte infiltration, disseminated intravascular coagulation, destruction of blood vessels, and hemorrhage (6). This can present in two clinical forms: cutaneous loxoscelism with classic dermonecrotic action and visceral or systemic loxoscelism with a hemolytic and procoagulant effect, the latter presents a smaller proportion of total cases (7) but is potentially fatal because it can cause disseminated intravascular coagulation (DIC) or multiple organ damage (DOM) leading to the patient´s death, being the systemic form the most frequent in children (8).

Antibiotics and steroids are commonly used in clinical practice, but it is not clear whether they have any beneficial effects in the treatment of loxoscelism, so there is currently no specific treatment for severe loxoscelism (9).

There are studies proving the efficacy of polyclonal antivenoms to treat loxoscelism clinical picture due to their Abs antigen-binding activity and neutralizing capacity (10). However, it is not very accessible to most of the population and there is doubt about the real neutralizing power of the cutaneous and systemic effects of the poisoning and the ideal period for its administration (11) due to lost effectiveness when administered late and the treatment is considered expensive. Molecular mechanisms of the poison are mediated by the enzyme Sphingomyelinase D, at the systemic level, it causes a severe inflammatory reaction, which ends up causing multiple organic damages, the kidney (7) and the lung being the most affected. In addition, there is an increase in oxidative stress which can culminate in the death of the patient. Therefore, in this work, we propose the use of hyperbaric oxygenation which has shown beneficial effects against the disease (12, 13); where adequate levels of pressure (less than 3 absolute atmospheres) and oxygen concentration are maintained, the risk of presenting any damage due to hyperbaric therapy is very small, such as middle ear barotrauma which is the most common side effect, but it is possible to prevent it effectively by the patient using Valsalva maneuvers; such as: swallowing, chewing, or trying to create positive pressure by blowing air (14). And N-acetylcysteine has shown efficacy in modulating inflammatory and oxidative stress factors in multiple similar pathologies (15, 16) This will allow us to offer a range of affordable treatments for vulnerable people, reducing morbidity and mortality from visceral loxoscelism.

## Materials and Methods

***Animals***

Male Wistar rats of 45 days of age, provided by the vivarium of the Escuela Superior de Medicina (Instituto Politécnico Nacional, Mexico City, Mexico), were kept in acrylic cages and randomly distributed into 5 different groups (n=5) for functional, morphological, and molecular studies: (group 1) Control: healthy without any treatment. (group 2) VLoxo: intradermal inoculation loin and no treatment. (group 3) HBO: with venom inoculation and hyperbaric chamber treatment. (group 4) NAce: venom inoculation and N-acetylcysteine treatment. (group 5) Mix: with venom inoculation and both N-acetylcysteine treatment and hyperbaric oxygenation therapy. Animals were acclimatized to laboratory conditions, including a 12 hr light/dark cycle (12:12). Sterile food and water were provided ad libitum. Animal handling by following the Mexican Federal Regulations for Animal Experimentation and Care (NOM-062-ZOO-1999, Ministry of Agriculture, Mexico City, Mexico) and approved by the Institutional Animal Care and Use Committee (CICUAL-ESM-IPN).

***Venom inoculation***

*Loxosceles boneti* venom was provided by UMA OCTOLAB with registration SEMARNAT-UMA-IN- CR-0086-VER/08 which was kept at -20 degrees and will be thawed 5 min before administration.

One day before inoculation the rat was shaved on the back to visualize more easily the inoculation area. It was administered intradermal inoculation on the loin (3 microliters/gram weight), and 30 min after the inoculation, the corresponding groups started the corresponding treatment.

The treatments were administered as follows:

Group 3: 200 milligrams per rat of NAce every 24 hr, the drug was dissolved in 1 ml distilled water and then proceeded for intraperitoneal administration.

Group 4: Hyperbaric Oxygen Therapy (HBO) one session every 24 hr at 2 absolute atmospheres for 1 hr: the animals were placed in a clean box with a plastic lid, inside the chamber, oxygen was supplied until reaching 2 total atmospheres and left inside the chamber for one hour.

Group 5: Mix (NAce/HBO) was administered NAce at the indicated dose and then subjected to HBO under the aforementioned conditions. All therapies were administered for 7 days. 

***Euthanasia***

The rats were euthanized after anesthesia with pentobarbital (60 mg/Kg/IP).

Blood samples were taken directly from the aortic arch and sent to the specialized veterinary laboratory (CEDIVETE Centro de Diagnóstico Veterinario Especializado, www.cedivete.com.mx) for analysis of blood biometry, liver function tests, and coagulation tests.

***Molecular analysis (Western blot)***

One hundred mg of kidney and lung tissue were taken for each study group previously collected and kept frozen at -80 ^°^C, homogenized in polytron in Tris solution, pH 7.4 with protease and phosphatase inhibitors (mini Complete Cocktail). They were centrifuged at 4 ^°^C at 10,000 G for 15 min and protein quantification was performed by the Bradford technique. A semi-dry Western blot was used to determine variations in COX1 and COX2. Subsequently, 100 µg of total protein was taken from each sample and separated by 10% SDS-PAGE gels under reducing conditions. They were then transferred to a polyvinylidene difluoride membrane. (Immobilon PVDF, 0.45 µm; Millipore, USA). The membranes were then blocked with 5% bovine serum albumin in TBS -0.1% Tween 20 (TBS-T, pH 7.4) for 2 hr, washed three times with TBS-T, and incubated with primary antibodies listed in Table 1 at 4 ^°^C overnight under continuous agitation. Detection was performed by using the enhanced chemiluminescence method (Western transfer luminol reagent, Santa Cruz Cat. 2048).

Membranes were photographed and the image was digitized to perform densitometric analysis using Image Studio Lite software (LI-COR Biosciences). The relative presence of each protein was normalized with β-actin as the housekeeping protein. The antibodies used were: primary antibodies: COX 1 Anti-rabbit Pro-sci 5391, (1:2000); COX2 Anti-rabbit Pro-sci 5393, (1:2000), Β actin Anti-Goat sc-1615, (1:3000); Secondary antibodies: Anti-Rabbit (Invitrogen) 656120, (1:6000); Anti-Goat (Invitrogen) 81-1620, (1:12500).

***Oxidative stress***

Animals were sacrificed immediately after the respective procedure using diethyl ether anesthesia, which affects oxidant/anti-oxidant parameters to a lesser extent compared with other procedures. The lung and kidney were removed and stored at -80 ^°^C, awaiting analysis. Samples were obtained by placing tissues in 30 mmol of cold phosphate buffer (pH 7.2) and adding 0.1% Triton 100 (1 mg of tissue per 10 μl of buffer). Tissues were homogenized and centrifuged at 10 000 rpm for 15 min (4 ^°^C) and supernatants were stored at -80 ^°^C for no more than two weeks before analysis.

In tissue homogenates, Cayman Chemistry (USA, MI) kits were used for the measurement of total protein (TP, No.704002), SOD (with assay Kit Cayman chemical kit No 706002), and CAT (CAT assay kit, No. 707002). In tissues that had a significant presence of this measurement in the lung and kidney, homogenates were treated with an Amicon Ultra-0.5 (30K) centrifugal filter device before quantification. The level of 3-nitrotyrosine (3NT, 3-Nitrothyrosine Elisa Kit, Abcam, No. ab116691, UK) was established in homogenates by enzyme-linked immunosorbent assay. SOD and CAT values are expressed as nmol/mg, and 3NT values are expressed as nmol/mg PT.

***Histological studies***

At the end of the treatments, all animals in each group were euthanized and their kidneys and lungs were dissected and fixed by immersion in 37% formaldehyde for 24 hr. Subsequently, the samples were washed profusely, processed with a conventional histological technique for embedding in paraffin, and sectioned into 7 μm thick sections. They were stained with hematoxylin and eosin and Hart’s stain for elastic lamelles. The sections were examined under a Zeiss microscope with a NIKON Eclipse E600 digital capture device.

***Statistical analysis***

Data are presented as mean±SEM. A comparison between the study groups was analyzed using the one-way ANOVA test with its subsequent Tukey posthoc test considering significant data with a *P*<0.05. All analyses and graphs were performed with GraphPad Prism version 8.0 software.

## Results

At the time of inoculation, respiratory distress, hypoactivity, bristling of the hair, and inappetence were observed. These signs were maintained until the moment of euthanasia in the group with poison and showed a tendency to recovery in the groups with treatment.

A blood smear was performed 5 days after the poisoning and it was observed that in the poisoned group there were abundant platelets and increased erythrocyte size. In the HBO group, the erythrocytes return to normal size, and in the NAce group, platelet clusters and increased erythrocyte size were observed. It should be noted that with both HBO and NAce, no increase in platelets was observed. However, in the combination of treatments there is no increase in platelets, erythrocyte size appears normal in most erythrocytes, but some are still increased.

**Figure 1:**
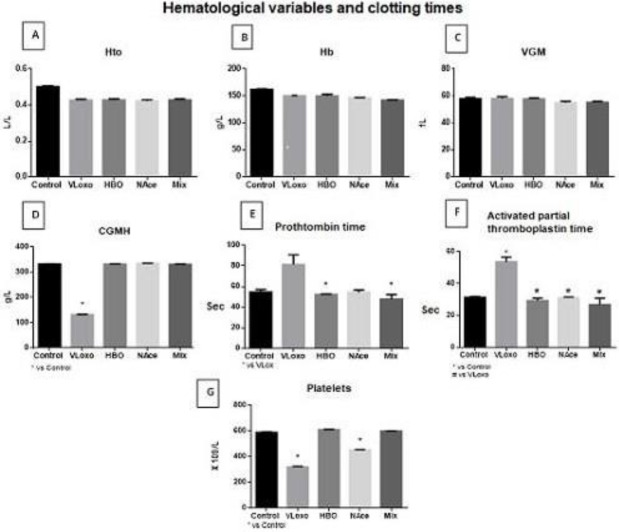
The image shows a hemogram obtained from different study groups; control, VLoxo, HBO, NAce, and Mix. A. Hematocrit test (Hto). B. Hemoglobin test (Hb). C. Mean globular hemoglobin (VGM). D. Mean globular hemoglobin concentration (MGHc) * Vs Control E. Prothrombin time. * Vs VLoxo. F. Activated thromboplastin time. *vs Control, # vs VLoxo. G. Platelet count * vs Control. * *P<*0.05 vs Control, *Loxosceles boneti* without treatment (VLoxo), *L. boneti* with HBO treatment (HBO), *L. boneti* with N Acetylcysteine treatment (NAce), with the combination of both treatments (Mix)

On the other hand, a hemogram was performed on the rats of the different groups, showing a slight decrease in Hto, Hb, erythrocytes, and VGM, but without reaching significance. It was also observed that in the poisoned group there was a significant decrease in corpuscular globular hemoglobin. This may explain the increased erythrocyte size in the blood smear. The previous results are criteria to diagnose disseminated intravascular coagulation, so in this model, it can be considered the existence of this. It should be noted that the treatments used tend to contribute to improving these variables. The state of disseminated intravascular coagulation characteristic of Loxosceles poisoning especially systemic damage is confirmed, which are increased prothrombin time, increased activated partial thromboplastin time, and decreased platelets due to its use. In addition, it is observed that the treatments used help to improve these variables (Figure 1).

A significant decrease in corpuscular globular hemoglobin and the index of hemoglobin contained in each erythrocyte is observed in the poisoned group. This may explain the increased erythrocyte size in the blood smear.

**Figure 2:**
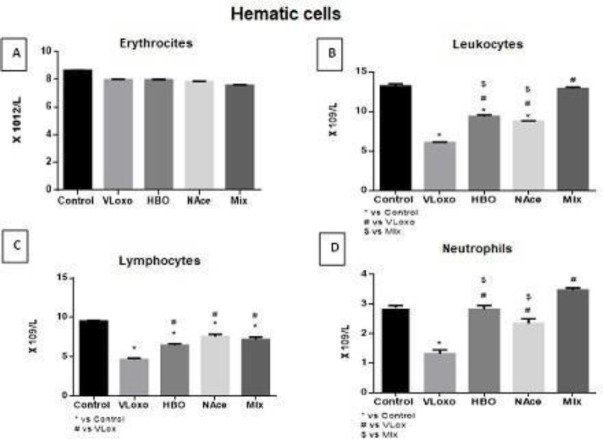
The image shows the cellular fraction of the hemogram obtained from the different study groups; control, VLoxo, HBO, NAce, and Mix; A. Erythrocytes. B. Leukocytes (x 109 /L). C. Lymphocytes (x 109 /L). D. Neutrophils (x 109 /L). A. p=ns, B. p=*vs Control, #vs VLoxo, $ vs Mix, C. p=*vs Control, #vs VLoxo, D. p=*vs Control, #vs VLoxo, $ vs Mix

A decrease in leukocyte species was observed in the groups with poison that were not subjected to any treatment, and interestingly, it should be noted that the groups with NAce and HBO treatment contributed to the recovery of these for both treatments, highlighting that the combined treatment is where an even more noticeable beneficial change is produced, an interesting aspect that can be observed is that both treatments together there is a slight recovery in leukocytes derived from neutrophils, being more noticeable when combining treatments, however, the lymphocyte line does not reach the basal state (Figure 2).

The animals were euthanized seven days later, and the area of the skin where the inoculum was located was removed, where an area of scarring in the fascia could be seen. It can also be observed that the animals that were inoculated with Loxosceles venom had visible damage in several tissues in the groups with poisoning, mainly in the lungs and kidneys. This damage was observed to a lesser degree in the groups that received treatment.

***Histopathological studies***

Representative histological sections at 20 and 40 X of the different study groups are shown. In the group that was administered the venom we observed evident destruction of the stellate lumen of the bronchiole in addition to rupture of alveolar atria and the presence of large thrombi in the pulmonary blood vessels; we also observed an increase in cellularity at the expense of leukocytes and thickening of alveolar walls.

**Figure 3:**
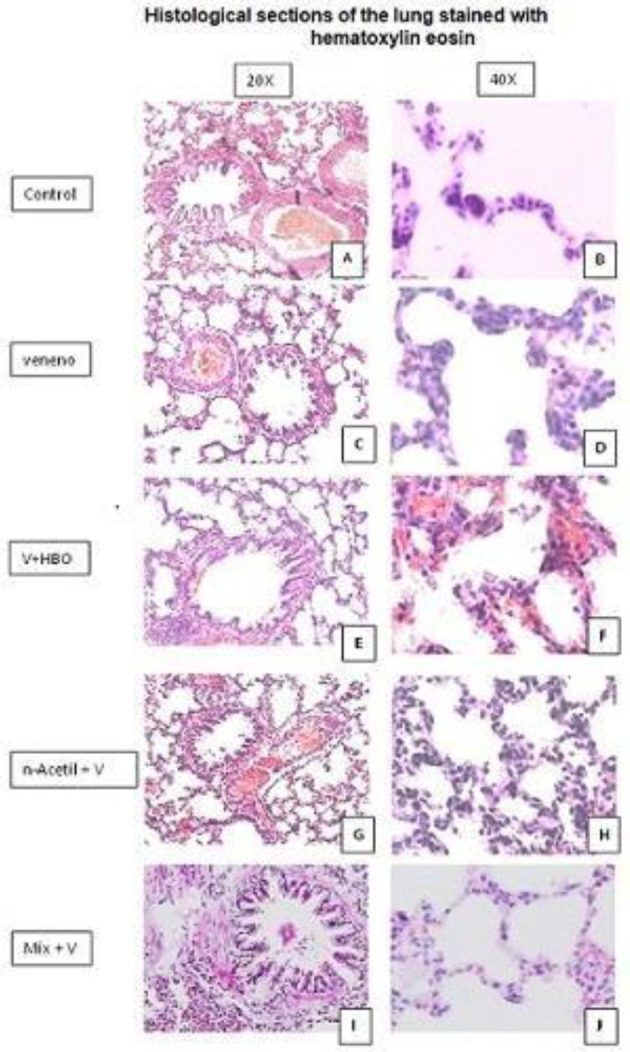
The image shows representative histological sections obtained from lung stained with Hematoxylin Eosin from the different study groups; control, VLoxo, HBO, NAce, and Mix at 20X (left) and 40X (right). A control group (A and B) is presented to analyze the bronchus and alveolar septa

In the treatment with HBO we observed, restoration of the stellate lumen of the bronchiole, a decrease in the rupture of the alveolar septa, and a decrease in cellularity in most of the tissue, however, in isolated areas, especially in peripheral areas of the lung, we still observed the strengthening of the alveolar septa and the presence of small intra-alveolar thrombi. A control group (A and B) is presented to analyze the bronchus and alveolar septa, which are observed with pathological changes at the bronchial level, besides the presence of thrombi of important size, also the destruction of the alveolar septa, being more notorious in the VLoxo group (C and D), when administering the different treatments, we observed a partial improvement in the integrity of the tissues, however in the HBO group (E and F), the presence of thrombi remains, although the inflammatory infiltrate diminishes the Nace group (Gand H) the presence of thrombi and thickening of the alveolar wall is observed, but with stability in the alveolar septa, in the Mix group (I and J), a greater recovery is observed at the bronchial level and decrease of the inflammatory infiltrate at alveolar level, with the presence of thrombi of smaller size. Therefore, we can conclude that at the morphological level the Mix group presented the greatest improvement (Figure 3).

**Figure 4:**
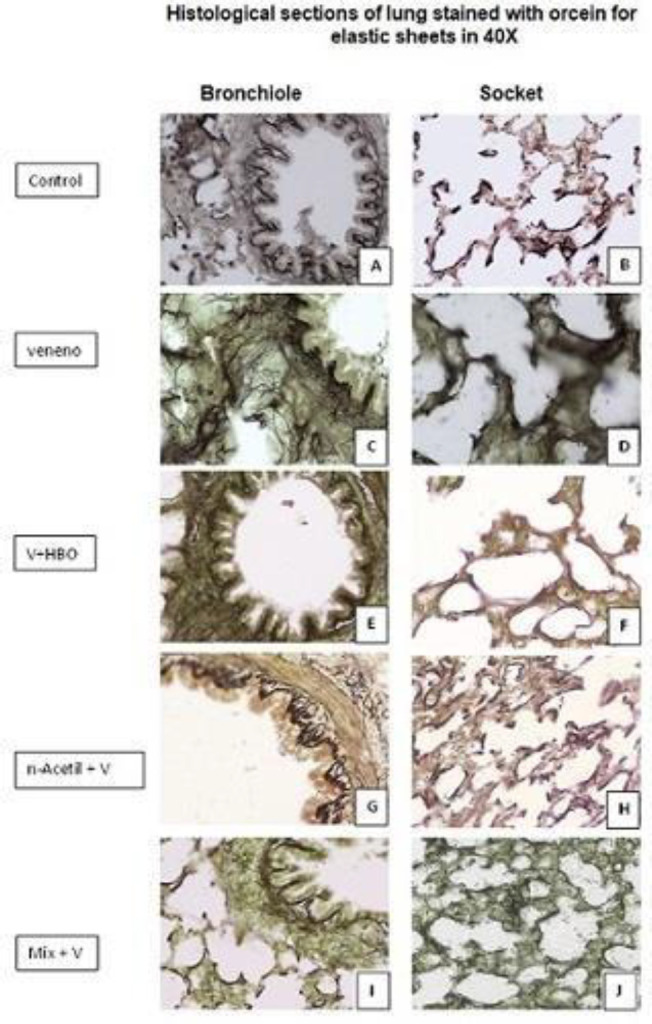
The image shows representative histological sections obtained from lung stained with acid fuchsin for elastic lamellae of the different study groups; control, VLoxo, HBO, NAce, and Mix at 20X (left) and 40X (right); A control group (A and B) is presented

In treatment with NAce, we observed a similar recovery of the stellate lumen of the bronchiole, but the thickening of the alveolar septa did not decrease. A control group (A and B) is presented, where we observe the elastic lamellae present at bronchial and alveolar levels, which run circumferentially to the structures, while in VLoxo (C and D), these lamellae are broken and disorganized, finding a thickening at the expense of fibrous and inflammatory material, in the HBO group (E and F), the organization of the lamellae is partially restored, some elastic lamellae remain fragmented and thickening at the expense of inflammatory material persists, in the Nace group (G and H), thickening of the elastic lamellae is observed, but there is a restoration of the organization, in the Mix group (I and J), a greater organization of the elastic lamellae is observed and thickening of the alveolar wall is no longer present, although bronchial thickening persists (Figure 4).

**Figure 5:**
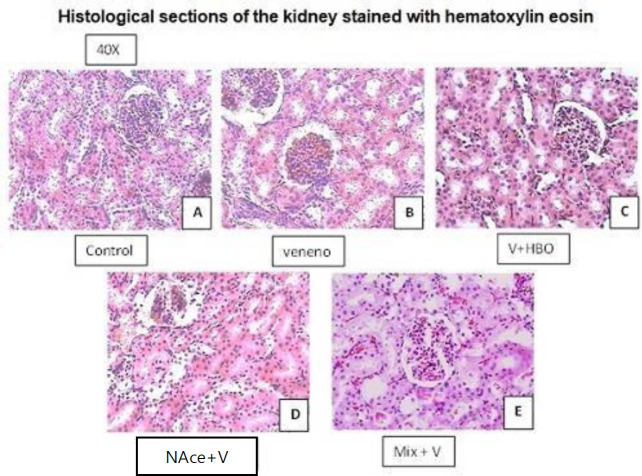
The image shows representative histological sections obtained from rat kidneys stained with Hematoxylin Eosin from the different study groups, visualized at 40X

For the kidney, representative histological sections at different magnifications of the study groups are shown. It can be observed that in the group to which the venom was administered there is a loss of the chorionic ratio of the medulla as well as a reinforcement of the cortex that may be due to the presence of leukocytes dispersed throughout the parenchyma, observed at 20 X. The glomerulus is congested and swollen. A control group (A) shows the renal corpuscle with integral contoured ducts and without lesions, the dense macula is integrated, while in the VLoxo group (B) glomerular lesión is observed, with increased cellularity and erythrocyte accumulation. In the HBO group (C), recovery of the structure of the glomerulus is observed, however, the increased cellularity continues and there is inflammation of the contoured ducts. In the group of Nace (D), an important glomerular lesión is observed, but the cellularity increases have diminished, the contoured ducts are seen integral. In the Mix group (E), the glomerular lesion is observed, but there is no increased cellularity, neither inflammatory infiltrate, there is slight edema at the level of contoured ducts and dense macula. We can conclude that at the renal level the damage remains despite both treatments (Figure 5).

These changes can also be observed in the treatments, with the difference that in both treatments the presence of leukocytes in the parenchyma is not found, but the glomeruli remain damaged. In N- acetylcysteine, we also have a reduction of the cortical surface.

***Oxidative stress***

**Figure 6:**
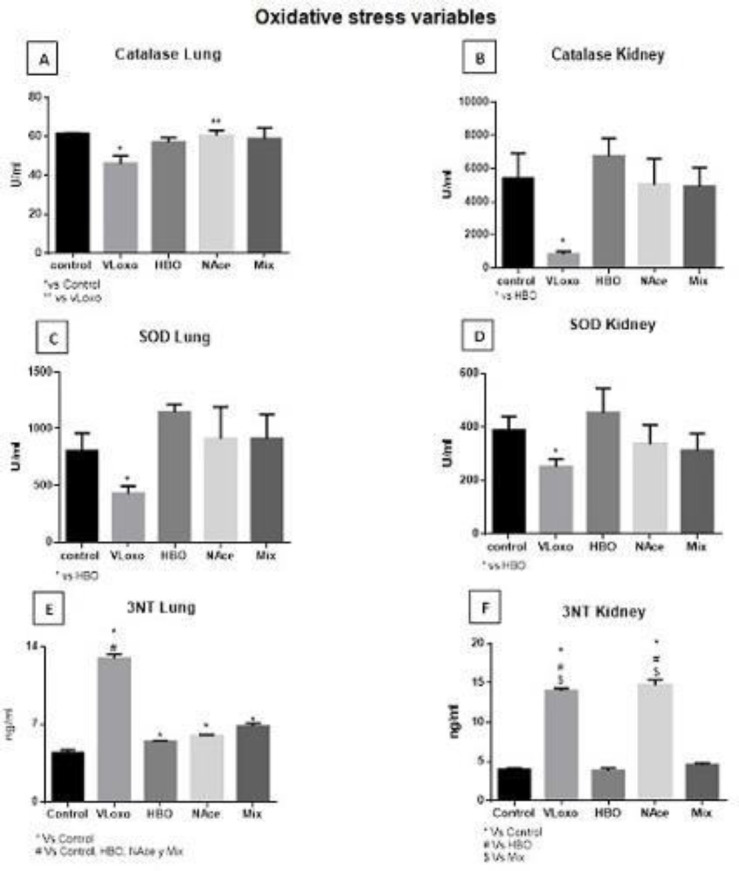
The image shows the analysis of the oxidant-antioxidant balance of the different study groups; control, VLoxo, HBO, NAce, and Mix. Quantified by Cayman^®^ Kit. 540 nm. Means±SEM (n=4 rats) are presented; image A) shows catalase in the lung (CAT), p=* Vs Control, p=** Vs VLoxo. Image B) shows CAT in the rat kidney, we observe a severe decrease in the VLoxo group p=* Vs HBO. Image C) shows superoxide dismutase in the lung (SOD), P=* Vs Control, HBO. In image D) kidney SOD, p=*Vs Control; Vs Control, HBO, Nace, and Mix. Image F shows 3Nt in the kidney vs HBO and Vs Mix

The analysis of the oxidant-anti-oxidant balance of the different study groups; control, VLoxo, HBO, NAce, and Mix shows a decrease of catalase in the lung (CAT) in the Vloxo group, but with the treatments, total recovery is found, the NAce group being the one that has a better recovery than the other groups. On the other hand, a decrease of CAT in the Kidney was observed in the VLoxo group, having a considerable recovery with the different treatments until reaching the basal state. Similar results were observed with superoxide dismutase in the lung and kidney(SOD), where we also observe a decrease in VLoxo, and in the treatments we have a recovery, the HBO group being the most recovered in the lung while in the kidney we observe again the decrease of this enzyme in VLoxo, having a recovery in the different treatments, being more important this recovery in HBO. A significant increase in tri-nitrotyrosine in the lung (3Nt) is observed in VLoxo and recovery in all treatments without reaching the basal state; while in the kidney, a significant increase is observed in VLoxo, and recovery to basal levels of HBO and Mix, but in Nace no changes are observed. We can conclude that both treatments improve oxidative status (Figure 6).

***Western blot***

**Figure 7:**
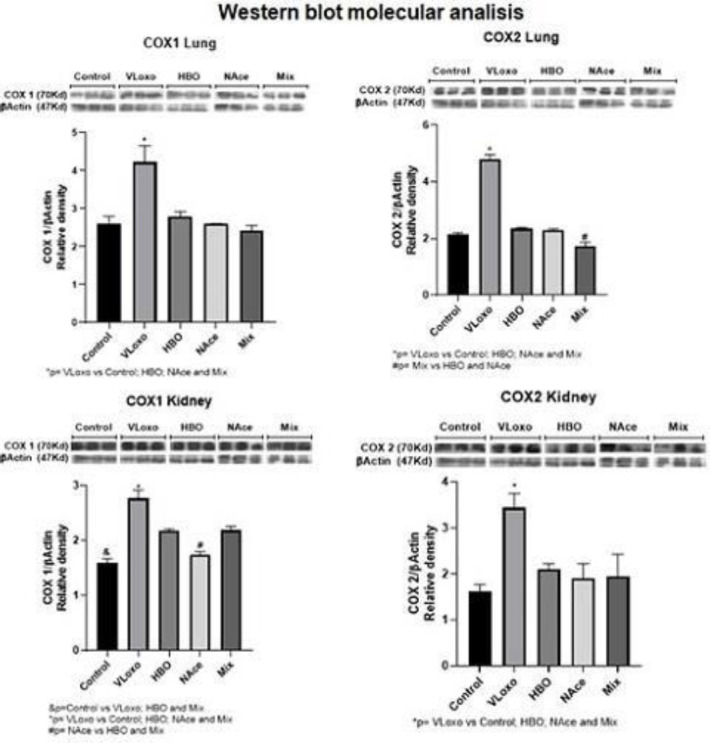
The image shows the molecular analysis by Western Blot for COX1 and COX2 proteins in the lung and kidney of rats from the different study groups: control, VLoxo, HBO, NAce, and Mix. The means±SEM are presented (n=4 rats). In image A) A significant increase in COX1 expression is observed in the VLoxo group and recovery in all treatments to basal state, p*=VLoxo vs Control; HBO, Nace and Mix in image B) shows a significant increase in COX2 expression in the lung, similar to COX1, with the same recovery effect of the different treatments, the Mix group being the least effective since it does not return to the basal state, p*=VLoxo Vs Control; HBO; Nace and Mix #=Mix vs HBO and NAce. Figure C shows the expression of COX1 in the kidney, and also shows a significant increase of COX1 in the kidney, although the treatments initiate a recovery, only the Nace group reaches the basal state, in the image, p&= Control vs VLoxo; HBO and Mix. *VLoxo vs Control; HBO; NAce and Mix. #=NAce vs HBO and Mix. D) shows the expression of COX2 in the kidney, where we can see a significant increase in expression in the VLoxo group p*=VLoxo vs Control; HBO; NAce and Mix, and restoration in the treatment groups, we can conclude that the treatments are effective at the molecular level to reduce inflammation, inhibiting the overexpression of these pro-inflammatory enzymes

The results show the molecular analysis by Western Blot for COX1 and COX2 proteins in the lung and kidney of rats from the different study groups: control, VLoxo, HBO, NAce, and Mix. In image A) A significant increase in COX1 expression is observed in the VLoxo group and recovery in all treatments to the basal state. Image B) shows a significant increase in COX2 expression in the lung, similar to COX1, with the same recovery effect of the different treatments, being the least effective in the Mix group since it does not return to the basal state. Figure C) shows the expression of COX1 in the kidney, and also shows a significant increase of COX1 in the kidney, although the treatments initiate a recovery, only the Nace group reaches the basal state. Figure D shows the expression of COX2 in the kidney, where we can see a significant increase in expression in the VLoxo group p*=VLoxo vs Control; HBO; NAce and Mix, and restoration in the treatment groups, we can conclude that the treatments are effective at the molecular level to reduce inflammation, inhibiting the overexpression of these pro-inflammatory enzymes (Figure 7).

VLoxo: Loxosceles venom; NAce: N-acetylcysteine; Mix: Loxosceles venom*HBO*NAce; HBO: Hyperbaric Oxygen Therapy

VLoxo: Loxosceles venom; NAce: N-acetylcysteine; Mix: Loxosceles venom*HBO*NAce; HBO: Hyperbaric Oxygen Therapy

VLoxo: Loxosceles venom; NAce: N-acetylcysteine; Mix: Loxosceles venom*HBO*NAce; HBO: Hyperbaric Oxygen Therapy

VLoxo: Loxosceles venom; NAce: N-acetylcysteine; Mix: Loxosceles venom*HBO*NAce; HBO: Hyperbaric Oxygen Therapy

HBO: Hyperbaric Oxygen Therapy

VLoxo: Loxosceles venom; NAce: N-acetylcysteine; Mix: Loxosceles venom*HBO*NAce; HBO: Hyperbaric Oxygen Therapy

VLoxo: Loxosceles venom; NAce: N-acetylcysteine; Mix: Loxosceles venom*HBO*NAce; HBO: Hyperbaric Oxygen Therapy

## Discussion

*L. boneti* venom was administered subcutaneously, showing its effects within a few min. The behavior of the rat changed and characteristic signs of envenomation were observed such as respiratory distress, hypoactivity, bristling of the hair, and lack of appetite (17, 18). These signs were maintained during the seven days before euthanasia, although not with the same intensity. When a systemic picture of Loxosceles developed, a hemogram was performed to follow up on the systemic poisoning, since it is common to find disseminated intravascular coagulation (9). With the results obtained in the hemogram, the criteria were met to consider that there is disseminated intravascular coagulation in the hemorrhagic phase (19).

Thrombocytopenia, prolongation of prothrombin time, and activated partial thromboplastin time were found, as well as erythrocyte abnormalities in the blood smear and a decrease in CGMH in the VLoxo group (20). This allows us to establish the criteria to consider DIC. These variables are reversed reaching states similar to the baseline in the treatment, HBO, Nace, and Mix groups.

For the cellular fraction of the blood, we found a decrease of platelets in the VLoxo group which strengthens the diagnosis of DIC by the use of thrombocytes, we have a reestablishment in the platelet count in the HBO and Mix groups but not in the Nace group. In the literature, it has been mentioned that N-acetylcysteine has a powerful anti-oxidant effect that could regulate platelet function, but not their quantity (Figure 1) (21). No modification is found in the erythrocyte count, however, in the erythrocyte count we have a decrease in VLoxo, having a partial restoration in the HBO and Nace treatments and a total restoration in the Mix treatment; this pattern is similar in both lymphocytes and neutrophils. This suggests an immunological regulation given by the treatments.

Histopathological, lung, and kidney sections, two of the most affected organs, were reviewed in the different study groups (22, 23). In the lung, destruction of the bronchial epithelium was observed in the V.Loxo group, with the formation of large clots and the presence of abundant leukocytes in the pulmonary artery, in addition to inflammatory thickening of the alveolocapillary wall with the destruction of emphysematous alveoli. In the HBO group, we observed restoration of the bronchial epithelium, preservation of the alveolar septa, decrease in the size of clots in the pulmonary artery, the persistence of alveolar wall thickening with parenchymal extravasation of erythrocytes. It is known that HBO therapy can increase vascularization and fibroblast proliferation which can be observed in this case (24). In the NAce group, restoration of bronchial epithelium and continuity of the alveolar wall, persistence of alveolar wall thickening, and persistence of clots in the pulmonary artery were observed. The combination of both treatments can be taken as an ideal result since there is an improvement in the lung parenchyma and a marked reconstruction of the bronchial epithelium. For the specific case of the lung, we can attribute the improvement in N-acetylcysteine treatment to the decrease in free radical damage (25).

The renal parenchyma in the V.Loxo group, HBO group, restoration of the cortex-medulla ratio, decrease of the glomerular inflammation, decrease of the parenchymal hemorrhage, and persistence of the dilatation of the collecting system, the structural preservation of the renal tissue is slightly reformed due to NAce, although it is not complete if it can be said that it helps, due to its anti-oxidant activity (27). The decrease in this damage is expected and has been demonstrated in other studies (26). In the NAce group, we observed restoration of the cortex-medulla ratio, persistence of glomerular inflammation, a decrease of parenchymal hemorrhage, and a decrease in the dilatation of the collecting system. the structural preservation of the renal tissue is slightly reformed due to NAce, although it is not complete if it can be said that it helps, due to its anti-oxidant activity (15).

In the lung elastic lamelles in the VLoxo group, there is a thickening of these, which indicates stiffness, in addition to pronounced disorganization and loss of direction compared with the control group, this is related to a severe inflammatory process that exists in this group, while for the groups treated as HBO the disorganization persists. Several studies show that there is a beneficial effect as well as the formation of collagen in the damaged tissue (27). It was observed in the NAce group that there is a slight recovery of the direction of the elastic lamellae, which is associated with the regulatory effect of this drug on oxidative stress (15). Similar results were observed in the Mix group, although they were not significant as in the groups where the treatments were separate. It should be noted that in this group there was collagen formation and reorganization of the elastic lamellae by the action of both treatments.

In the lung and kidney there is a decrease in the levels of the superoxide dismutase enzyme in the VLoxo group compared with the control group, and a significant recovery with treatments for both organs (15, 28).

Tri-nitrotyrosine (3Nt) is a metabolite-generated hyper oxidation regulated by eNOS and is elevated when there is significant oxidative stress. It is elevated in the lung and kidney in the VLoxo groups and is related to the damage observed in pathological studies; while in the HBO, Nace, and Mix groups the levels of 3Nt are restored to the basal state with the particular exception of Nace in the kidney, which does not show a decrease. This could indicate that hyperbaric oxygen has a more potent anti-oxidant effect than Nace and that there is no difference in their combination (29).

Cyclooxygenase 2 (COX-2) is increased in inflammation and diseases such as chronic obstructive disease so COX-2 protein can be shown in high levels in the parenchyma and airways so it is expected that these levels are related to the damage caused (30).

Consequently, we can see that in the results obtained we have the same elevation of cyclooxygenases COX2 and COX1 in the lung specifically in the VLox group where we know the most evident damage is found because it does not present any treatment results expected for this group. Interestingly, we observed that there is a significant decrease in the groups that received treatment of some kind, but highlighting the decrease of COX2 in the NAce group, as well as in the Mix group, this may be due to the effect they have on anti-oxidants to affect cyclooxygenases, and thus the inhibition of prostaglandin production (31).

This effect will act against the inflammation produced as a consequence of the administered poison. N-acetylcysteine acting as an anti-oxidant, in this case, affects the production of prostaglandins on monocytes activated by COX inhibitors, where it is known that there is a reduction of this due to administration of N-acetylcysteine, In addition to the reduction of free radical damage, something similar occurs with kidney where a decrease of COX 1 is observed in the N-ace and Mix groups, where previously there was an evident increase of COX 1 and 2 in the VLOX group (31).

## Conclusion

The results obtained in the present work provide us with evidence suggesting that the proposed alternatives for the treatment of systemic Loxoscelism in the acute phase are safe and effective and that they can improve the patient’s prognosis. Both HBO and N-acetylcysteine showed an evident improvement in the systemic damage both at an oxidative and inflammatory level and having a morphological recovery in the kidney and lung; however, it is necessary to continue with investigations that allow us to determine the best long-term recovery therapy, to be able to offer patients safe, effective and affordable options that help to save lives, especially in children, who are the most affected.

## Authors’ Contributions

MTB and AFV helped with development, research, and writing of the paper; SASV and ADR performed histopathological studies; GGB and PLS performed toxicological studies; AKK performed oxidant and anti-oxidant studies; RAJL performed histopathological studies; LPE helped translate and review; MCCH was responsible for development, analysis, writing, and direction of the project.

## Conflicts of Interest

The authors have no conflicts of interest and declare that this article has not been published nor is being considered for publication elsewhere.
